# Surgical Site Infection by *Corynebacterium macginleyi* in a Patient with Neurofibromatosis Type 1

**DOI:** 10.1155/2013/970678

**Published:** 2013-05-30

**Authors:** Bruno Cacopardo, Stefania Stefani, Francesco Cardì, Carlo Cardì, Marilia Rita Pinzone, Giuseppe Nunnari

**Affiliations:** ^1^Department of Clinical and Molecular Biomedicine, Division of Infectious Diseases, University of Catania, Via Palermo 636, 95125 Catania, Italy; ^2^Department of Microbiology, University of Catania, 95100 Catania, Italy; ^3^Department of Surgery, Transplantation and Advanced Technologies, General Surgery Unit, University of Catania, 95100 Catania, Italy

## Abstract

*Corynebacterium (C.) macginleyi* is a gram positive, lipophilic rod, usually considered a colonizer of skin and mucosal surfaces. Several reports have associated *C. macginleyi* with ocular infections, such as conjunctivitis and endophthalmitis. However, even if rare, extraocular infections from *C. macginleyi* may occur, especially among immunocompromised patients and patients with indwelling medical devices. We report herein the first case of surgical site infection by *C. macginleyi* after orthopaedic surgery for the correction of kyphoscoliosis in a patient with neurofibromatosis type 1. Our patient developed a nodular granulomatous lesion of about two centimetres along the surgical scar, at the level of C4-C5, with purulent discharge and formation of a fistulous tract. Cervical magnetic resonance imaging showed the presence of a two-centimetre fluid pocket in the subcutaneous tissue. Several swabs were collected from the borders of the lesion as well as from the exudate, with isolation of *C. macginleyi*. The isolate was susceptible to beta-lactams, cotrimoxazole, linezolid, and glycopeptides but resistant to quinolones, third-generation cephalosporins, and erythromycin. Two 30-day courses of antibiotic therapy with amoxicillin/clavulanate (1 g three times/day) and cotrimoxazole (800/160 mg twice a day) were administered, obtaining a complete healing of the lesion.

## 1. Introduction

*Corynebacterium (C.) macginleyi* is a gram positive, lipophilic rod, first identified in 1995 by Riegel et al. [1]; as other nondiphtheria *Corynebacterium* species, commonly termed diphtheroids, it is usually considered a colonizer of skin and mucosal surfaces [2]. However, several reports have shown *C. macginleyi *to be associated with ocular infections, such as conjunctivitis and endophthalmitis [3, 4]. Nowadays, there is evidence that *C. macginleyi* may be responsible for extraocular infections in immunocompromised patients and patients with indwelling medical devices. In 2002, Villanueva et al. described the first case of nonconjunctival infection with *C. macginleyi *in an old man with a permanent bladder catheter and vesical stones [5]. Subsequent reports identified *C. macginleyi* as the causative agent of endocarditis [6, 7], septicaemia [8], and intravascular catheter-associated bloodstream infection [9, 10]. In a recent report of Dias et al., *C. macginleyi *was repeatedly isolated from tracheostomy secretions of a patient with laryngeal carcinoma [11].

We report herein the first case of surgical site infection by *C. macginleyi *after orthopaedic surgery for the correction of kyphoscoliosis in a patient with neurofibromatosis type 1.

## 2. Case Presentation

In April 2008, a 14-year-old male patient, affected with neurofibromatosis type 1, underwent instrumented posterior spinal arthrodesis of T2–T10 because of severe cervicothoracic kyphoscoliosis. Posterior spinal arthrodesis was extended to C6–T2 when he was fifteen, due to worsened scoliosis. Eight months after surgical intervention, a nodular lesion of about two centimetres appeared along the surgical scar at the level of C4–C5. There were no signs of inflammation, and the nodule spontaneously disappeared after few days. 

**Figure 1:**
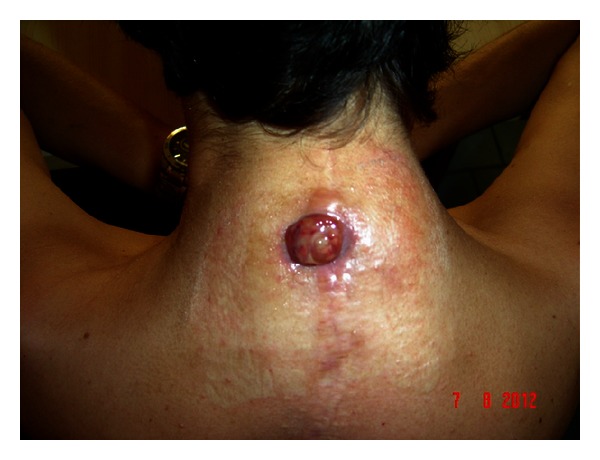
Formation of a round-shaped granuloma of about 2 centimetres along the surgical scar from a previous posterior spinal arthrodesis for kyphoscoliosis, in a patient with neurofibromatosis type 1. The granuloma evolved from a nodular lesion, which spontaneously drained, with purulent discharge and formation of a fistulous tract.

**Figure 2:**
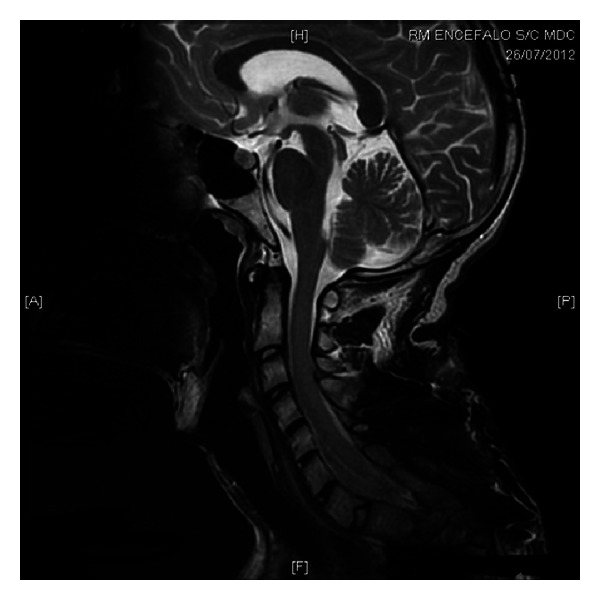
Cervical magnetic resonance imaging showing the presence of a fistulous tract and a fluid pocket in the subcutaneous tissues overlying C4.

In June 2012, a painful, tender and swollen lesion reappeared at the same site; it spontaneously drained, with purulent discharge and formation of a fistulous tract. The lesion evolved towards a round-shaped granuloma of about 2 centimetres (Figure 1). Cervical magnetic resonance imaging showed the presence of a two-centimetre fluid pocket in the subcutaneous tissue (Figure 2).

Several swabs were collected from the borders of the lesion as well as from the exudate, with isolation of *C. macginleyi. *Identification was performed by biochemical tests (API-Coryne system; bioMérieux SA, Marcy-l'Etoile, France) and confirmed by the sequencing of an internal fragment of the 16S rRNA gene [12]. Antibiotic susceptibility was evaluated by broth microdilution and agar diffusion methods, following EUCAST guidelines for alpha-hemolytic streptococci (http://www.escmid.org/). The isolate was susceptible to beta-lactams, cotrimoxazole, linezolid, and glycopeptides but resistant to quinolones, third-generation cephalosporins, and erythromycin.

**Figure 3:**
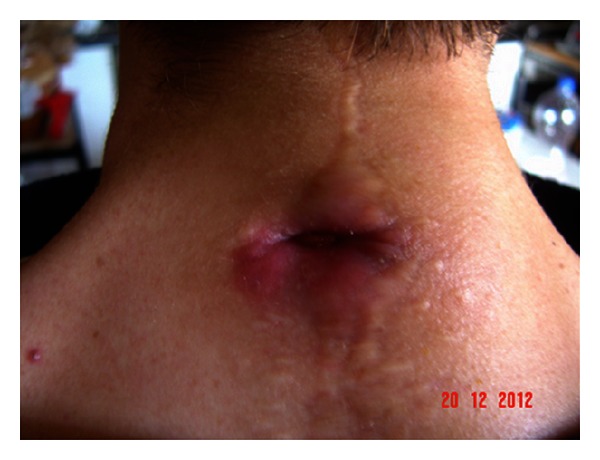
Significant reduction of the granuloma size after a thirty-day antibiotic treatment with amoxicillin/clavulanate and cotrimoxazole.

Considering the susceptibility profile of the isolated strain, oral antibiotic treatment with amoxicillin/clavulanate (1 g three times/day) and cotrimoxazole (800/160 mg twice a day) was administered for 30 days. The granulomatous lesion significantly reduced in size as well as the purulent discharge (Figure 3). In January 2013, the patient received a second course of antibiotics with the same schedule: amoxicillin-clavulanate (1 g three times/day) and cotrimoxazole (800/160 mg twice a day) for 30 days, achieving the complete healing of the lesion. At 3-month followup, the scar was well healed, with no signs of infection.

## 3. Discussion 

*C. macginleyi* has been usually isolated from ocular surfaces [2–4] and rarely associated with extraocular infections [5–11]. To the best of our knowledge, we describe the first case of surgical site infection caused by *C. macginleyi. *Although corynebacteria other than *C. diphtheriae* are usually considered skin and mucosal colonizers, in our case the isolation of *C. macginleyi *in pure culture from several samples suggested that it may have had a causative role.

In the present case report, the patient developed a soft tissue infection in the site of a previous surgical intervention. It may be hypothesized *C. macginleyi *to act as an opportunistic pathogen, possibly inoculated into the subcutaneous tissues during surgery, which may have found there the most favorable environment. In fact, moist regions seem to be the preferred habitat for diphtheroids [1]. Inflammation, which may occur after surgical trauma, might, therefore, favor the proliferation of these bacteria. 

In addition, *C. macginleyi *has been reported to form biofilm [13]. In previous case reports, *C. macginleyi* was identified as the pathogen causing infections associated with intravenous and bladder catheters [5, 9, 10]. The association of *C. macginleyi* infections with the presence of prosthetic abiotic materials suggests that biofilm formation may contribute to its virulence. However, little is known about the mechanisms of biofilm formation by corynebacteria, and further studies should address this topic.

A variety of antibiotic regimens have been used in the management of extraocular infections by C.* macginleyi. *In a recent report from Japan, *C. macginleyi* ophthalmic isolates showed high-level fluoroquinolone resistance [14], which was confirmed in all the published cases of extraocular infections [5–11], including the present report. The susceptibility of the isolated strains to other antibiotic classes seems to be different: Dobler and Braveny [9] isolated a strain resistant to a great number of antibiotics (quinolones, beta-lactams, beta-lactams with inhibitors, monobactams, gentamicin, macrolides, cotrimoxazole, and lincosamides) and sensitive only to vancomycin, netilmicin, and tetracycline. Other reports have shown a more favorable susceptibility profile, as the isolated strains were usually sensitive to beta-lactams, rifampin, gentamicin, and glycopeptides but generally resistant to quinolones and cotrimoxazole.

## 4. Conclusion

In conclusion, even if rare, extraocular infections by *C. macginleyi* may occur. The isolation of *C. macginleyi* in pure culture samples, especially among immunocompromised patients, should not be underestimated. As medical conditions that compromise the immune system become more widespread, the prevalence of *C. macginleyi *infections is expected to increase. 

Further research should better establish the pathogenetic mechanisms as well as predisposing factors to systemic infection.
